# Timing and severity of COVID-19 during pregnancy and risk of preterm birth in the International Registry of Coronavirus Exposure in Pregnancy

**DOI:** 10.1186/s12884-022-05101-3

**Published:** 2022-10-18

**Authors:** Louisa H. Smith, Camille Y. Dollinger, Tyler J. VanderWeele, Diego F. Wyszynski, Sonia Hernández-Díaz

**Affiliations:** 1 Department of Epidemiology, Harvard T.H. Chan School of Public Health, 677 Huntington Avenue, Boston, ME 02115 USA; 2 Roux Institute at Northeastern University, 100 Fore St, Portland, ME 04101 USA; 3 Pregistry, Los Angeles, CA USA

**Keywords:** SARS-CoV-2, Preterm delivery, Pregnancy outcomes, Viral infection, Case-time-control, Immortal time bias, Spontaneous preterm, Indicated preterm

## Abstract

**Background:**

Studies of preterm delivery after COVID-19 are often subject to selection bias and do not distinguish between early vs. late infection in pregnancy, nor between spontaneous vs. medically indicated preterm delivery. This study aimed to estimate the risk of preterm birth (overall, spontaneous, and indicated) after COVID-19 during pregnancy, while considering different levels of disease severity and timing.

**Methods:**

Pregnant and recently pregnant people who were tested for or clinically diagnosed with COVID-19 during pregnancy enrolled in an international internet-based cohort study between June 2020 and July 2021. We used several analytic approaches to minimize confounding and immortal time bias, including multivariable regression, time-to-delivery models, and a case-time-control design.

**Results:**

Among 14,264 eligible participants from 70 countries who did not report a pregnancy loss before 20 gestational weeks, 5893 had completed their pregnancies and reported delivery information; others were censored at time of their last follow-up. Participants with symptomatic COVID-19 before 20 weeks’ gestation had no increased risk of preterm delivery compared to those testing negative, with adjusted risks of 10.0% (95% CI 7.8, 12.0) vs. 9.8% (9.1, 10.5). Mild COVID-19 later in pregnancy was not clearly associated with preterm delivery. In contrast, severe COVID-19 after 20 weeks’ gestation led to an increase in preterm delivery compared to milder disease. For example, the risk ratio for preterm delivery comparing severe to mild/moderate COVID-19 at 35 weeks was 2.8 (2.0, 4.0); corresponding risk ratios for indicated and spontaneous preterm delivery were 3.7 (2.0, 7.0) and 2.3 (1.2, 3.9), respectively.

**Conclusions:**

Severe COVID-19 late in pregnancy sharply increased the risk of preterm delivery compared to no COVID-19. This elevated risk was primarily due to an increase in medically indicated preterm deliveries, included preterm cesarean sections, although an increase in spontaneous preterm delivery was also observed. In contrast, mild or moderate COVID-19 conferred minimal risk, as did severe disease early in pregnancy.

**Supplementary Information:**

The online version contains supplementary material available at 10.1186/s12884-022-05101-3.

## Background

Since its emergence, coronavirus disease 2019 (COVID-19) has proven uniquely harmful to certain populations [1, 2]. However, its effects on the pregnant population have been less easily discerned. Early studies suggested an elevated risk of preterm birth among pregnant people with COVID-19 [3–7] but were limited by small samples from single hospitals, little variability of disease severity or timing of infection during pregnancy, exclusion of ongoing pregnancies from estimates, and lack of valid comparison groups. More recent studies have reported a small but consistent increase in preterm delivery among people with COVID-19 during pregnancy [8, 9] or at delivery [10–12]. However, existing studies are limited in the extent to which they consider timing of infection; when gestational age at infection and start of follow-up are not aligned, “immortal time bias” may reduce, negate, or reverse any effect on prematurity [13]. In addition, although the daily rate of preterm delivery increases as week 37 approaches, the total risk of preterm delivery declines due to the shrinking window in which to deliver before term, making estimates of risk after infection at different gestational ages difficult to interpret. Finally, while associations between severe COVID-19 and preterm delivery may reflect biological effects of infection or the immunological response, they may also result from medically indicated delivery based on health concerns [5, 8, 11, 14]. No study has considered the timing of infection during pregnancy, the severity of disease, the indication of prematurity, and the methodological issues simultaneously.

Using data from a large, international pregnancy cohort, we investigated whether COVID-19 increased the risk of preterm birth. We used multiple analytic approaches to disentangle the role of severe disease and the timing of infection during pregnancy, allowing us to estimate gestational-age-specific risks and the respective roles of spontaneous and medically indicated preterm delivery after mild, moderate, and severe COVID-19 throughout pregnancy.

## Methods

### Cohort

The International Registry of Coronavirus Exposure in Pregnancy (IRCEP) began enrollment in June 2020 to participants in 10 languages (ClinicalTrials.gov identifier NCT04366986) [15]. Enrollees must be pregnant or within 6 months after pregnancy and have had a test for severe acute respiratory syndrome coronavirus 2 (SARS-CoV-2) or a clinical diagnosis of COVID-19 from a healthcare provider during pregnancy. A valid mobile phone number and internet access are required for enrollment, and recruitment was primarily via social media. Information is collected via online survey modules covering demographics, health history, COVID-19 symptoms and treatments, and pregnancy outcomes. Participants who join during pregnancy are sent reminders via text messages to complete monthly follow-up surveys; all participants receive messages encouraging completion of unfinished surveys. The Institutional Review Board of the Harvard T.H. Chan School of Public Health approved this study (IRB20–0622).

### SARS-CoV-2 infection and COVID-19

Participants reported dates, types (nose/throat swab for polymerase chain reaction (PCR) or blood test for antibodies), and results of SARS-CoV-2 tests during pregnancy, as well as clinical diagnoses, symptoms, and treatments of COVID-19 and their timing. We defined SARS-CoV-2-positive participants as those with a positive test or a clinical diagnosis. SARS-CoV-2-negative participants were those reporting only negative test(s) and no clinical diagnosis of COVID-19. Participants who enrolled during pregnancy were able to report additional tests later in pregnancy; if no positive test was reported, they were considered negative for the duration of pregnancy.

Following clinical guidelines for classifying COVID-19 severity [16], we considered anyone who was admitted to the intensive care unit (ICU), needed respiratory assistance (including ventilation or extracorporeal membrane oxygenation (ECMO)), or was hospitalized with reported organ failure, acute respiratory distress syndrome, pneumonia, an abnormal chest X-ray or CT scan, or indications of significant lung involvement to have had severe disease. Moderate infections were those with lesser lung involvement or other symptoms that resulted in use of health care outside of the home. Participants with other symptoms were considered mild, and those without reported symptoms, asymptomatic.

### Gestational age and delivery outcomes

Participants reported due dates as determined by last menstrual period and by ultrasound when available. Date of delivery and gestational age were reported upon pregnancy completion. We used these data to determine gestational age at COVID-19 symptom onset and test date(s), enrollment, and end of pregnancy.

Preterm birth was defined as delivery before 37 weeks’ gestation. We also collected information on mode of delivery, preterm labour, and premature rupture of membranes (i.e., before the onset of labour). We considered preterm delivery to be spontaneous if either spontaneous preterm labour or preterm premature rupture of membranes was reported, and medically indicated otherwise. The indicated category included cesarean sections performed before term when not preceded by spontaneous labor or membrane rupture. In a sensitivity analysis we analyzed very preterm birth using a cutoff of 34 weeks.

### Study sample

**Fig. 1:**
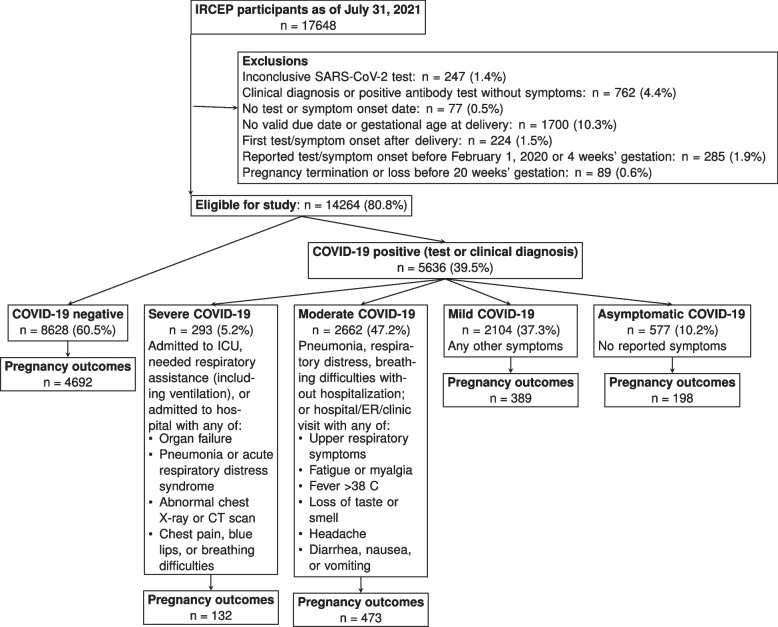
Participants in the International Registry of Coronavirus Exposure in Pregnancy (IRCEP) and their eligibility for this study

IRCEP participants as of July 31, 2021 who had not incurred early pregnancy loss (<20 weeks) or termination were eligible for our study (Fig. 1). We excluded those who reported COVID-19 before February 1, 2020 due to concerns about reporting error, as well as those for whom we could not estimate gestational age. In addition, we excluded those who reported no symptoms and a positive antibody test (i.e., infection cannot be timed) or inconclusive test results.

Participants who had not provided delivery information were excluded from analyses involving mode of delivery. In addition, we excluded from some analyses those missing data on baseline covariates. As sensitivity analyses we used multiple imputation to impute missing baseline covariates, used inverse-probability of censoring weighting to adjust for loss to follow-up, excluded those who had received a clinical diagnosis with no positive test, and conducted other robustness checks, which we report in Additional file 1.

### Statistical analysis

We compared baseline characteristics by COVID-19 status and severity and between those with and without outcome information. Among those with reported outcomes, we estimated unadjusted risk of preterm delivery, overall and separately for indicated vs. spontaneous deliveries. Because asymptomatic participants’ positive test dates were closely linked to their delivery dates (likely due to routine screening at delivery), artificially increasing the apparent risk of preterm delivery among asymptomatic infections close to 37 weeks, we excluded them from the remaining analyses [17].

### Preliminary analysis: multivariable regression

Among the participants whose pregnancies had ended, we regressed preterm birth on COVID-19 to estimate the relative risk of preterm birth after symptomatic COVID-19 at any time vs. never before 37 weeks of pregnancy. Participants with COVID-19 onset after 37 weeks’ gestation (no longer at risk for preterm birth) were excluded. We also excluded all participants whose last menstrual periods were within 45 weeks prior to the analysis date to allow sufficient time for term deliveries and avoid over-inclusion of shorter pregnancies. We fit the model using log-linear regression (Poisson regression with robust standard errors), adjusting for continent (Africa, Asia, Europe, North America, South America), maternal age (years), pre-pregnancy BMI (kg/m^2^), parity (primi−/multiparous), race/ethnicity (Asian, Black, Latina, White, mixed, other), pre-existing condition (chronic diabetes, asthma, cardiovascular disease, or autoimmune disease), healthcare coverage (yes/no), and reason for testing (symptoms, contact tracing, surveillance, other/not tested). To assess risks specifically due to severe COVID-19, we estimated relative risks of severe vs. mild or moderate disease among COVID-positive participants only (in sensitivity analyses, we separated mild and moderate disease to investigate any differences). Finally, we fit multinomial logistic regression models for a three-levelled outcome: spontaneous preterm, indicated preterm, and term delivery.

### Accounting for gestational age: time-to-delivery model

We improved upon the previous model by modelling time-to-delivery, which allowed us to use data from participants until they were censored, even if they never reported their delivery outcomes. To avoid immortal time bias, we compared participants who had COVID-19 symptom onset in a given week of gestation to those without COVID-19 who were still pregnant at that gestational age. Because risk of preterm birth differs by week of gestation even in the absence of infection, this approach allowed for the estimation of gestational age-specific risks and risk ratios (we also present risk differences in Additional file 1). Specifically, we asked the question, “What is the risk of preterm delivery in pregnancies affected by COVID-19 at week *x* of gestation, and how does it compare to the risk in pregnancies that are uninfected but ongoing at week *x*?”

First, for every week of gestation through week 36, we selected the individuals with COVID-19 that week (time zero) and, as comparators, all COVID-negative participants whose pregnancies were ongoing. COVID-negative participants could appear in repeated comparison groups until they delivered or were censored. Participants without outcomes were censored at the last known gestational week at which we knew their pregnancy was ongoing. We estimated daily hazards of delivery for each week-specific subcohort of symptomatic positive and test negative individuals from their time zero through week 42. To account for confounding and non-random censoring we fit a pooled logistic regression model, combining data from all subcohorts, with the previously described covariates, cubic splines for gestational age and week of infection, terms for time since infection and COVID-19 severity, and product terms for severity and time since infection.

To estimate the risk of delivery in each of the weeks following either mild/moderate, severe, or no COVID-19 at a given week of gestation, we used the predicted probabilities from the model to compute a standardized Kaplan-Meier estimator for each COVID-19/time zero combination. We used the baseline covariates in the COVID-negative participants to standardize survival for each of the COVID-19 groups. We then computed the overall probability of delivery any time before week 37. We combined estimates for infection before week 20 as there were no deliveries in those weeks and few infections. We estimated risks associated with any positive COVID-19 as a weighted average of the risks for mild/moderate and severe disease.

To estimate risk of spontaneous preterm delivery specifically, we fit a logistic regression model for spontaneous delivery among all preterm deliveries, conditional on gestational age at delivery, COVID-19 severity, weeks since infection, continent, pre-pregnancy BMI, parity, and race. We estimated risk of spontaneous preterm delivery as the probability predicted from that model multiplied by risk of any delivery at a given gestational age (the latter estimated as above).

We computed 95% confidence intervals using the non-parametric bootstrap. All analyses were conducted in R version 4.0.

### Robustness to unmeasured confounding: case-time-control design

The previous analyses assume confounders (i.e., risk factors for both preterm delivery and severe COVID-19) are measured; to reduce risk of unmeasured between-person time-fixed confounding, we conducted a within-person analysis using a case-time-control design [18, 19]. Although both the study population and the parameter being estimated are different from the primary analysis above, an association in this design would support the presence of effects. If COVID-19 has an acute, transient effect on preterm birth, among people who delivered preterm (i.e., those susceptible to prematurity), COVID-19 would have more likely occurred during the period in which it affected delivery timing (i.e., presumptively in the weeks before delivery) than earlier in pregnancy. We therefore compared, among preterm births (cases), the odds of symptomatic COVID-19 in the 30 days preceding delivery to a reference period 120–90 days prior, when we hypothesized it less likely to affect delivery. However, infection is not equally likely every week of pregnancy, so we made the same comparison among term births (controls) to estimate time trends, matching cases to controls on month of due date. Among controls we compared odds of any COVID-19 in gestational age windows corresponding to those of their matched cases, then divided out this time effect from the effect among the cases. This process is equivalent to fitting a conditional logistic regression for exposure (COVID-19) with indicators for case/control status (preterm vs. term delivery), risk/reference period, and their interaction, among a dataset with a row for each observation in the risk and reference period. To assess COVID-19 severity, we repeated the analysis using an indicator of severe disease as the exposure, and separately for mild/moderate disease (each compared to no COVID-19).

## Results

### Sample

Of 14,264 IRCEP eligible participants from 70 countries, 60.2% enrolled while pregnant (Fig. 1). Compared to individuals with negative tests, those with COVID-19 were of similar ages (30.1 vs. 30.6, among positive and negative, respectively) and had similar pre-pregnancy BMI (26.7 vs. 27.2). However, those participants with positive tests were more likely to be from South America (36.8% vs. 15.6%) and had fewer pre-existing conditions (12.0% vs. 15.2%) (Table 1); the same was true among the subset of participants providing pregnancy outcomes (Table S1 in Additional file 1).

**Table 1:** Descriptive characteristics^a^

	COVID-19 negative *N* = 8628	COVID-19 positive *N* = 5636	Total *N* = 14,264
Enrollment^b^			
Prospective	4033 (47%)	4555 (81%)	8588 (60%)
Retrospective	4595 (53%)	1081 (19%)	5676 (40%)
Prospective enrollees			
Follow-up data	243 (6.0%)	167 (3.7%)	410 (4.8%)
Weeks past LMP at enrollment (median (IQR))	27 (18, 35)	25 (17, 33)	26 (17, 34)
Weeks past LMP at symptom onset/test (median (IQR))	21 (12, 30)	19 (10, 27)	20 (11, 28)
Weeks past LMP at delivery or end of follow-up (median (IQR))	28 (12, 36)	26 (17, 33)	27 (18, 35)
Retrospective enrollees			
Follow-up data	4449 (97%)	1025 (95%)	5474 (96%)
Weeks past LMP at enrollment (median (IQR))	49 (44, 54)	47 (43, 53)	49 (44, 54)
Weeks past LMP at symptom onset/test (median (IQR))	38 (37, 39)	34 (28, 38)	38.0 (35.4, 39.3)
Weeks past LMP at delivery or end of follow-up (median (IQR))	39 (38, 40)	39 (38, 40)	39 (38, 40)
COVID-19 severity			
Negative	8628 (100%)		8628 (60%)
Asymptomatic		577 (10%)	577 (4.0%)
Mild		2104 (37%)	2104 (15%)
Moderate		2662 (47%)	2662 (19%)
Severe		293 (5.2%)	293 (2.1%)
COVID-19 diagnosis/test type			
Negative	8628 (100%)		8628 (60%)
Positive by antibodies only		531 (9.4%)	531 (3.7%)
Positive by throat/nose swab		4486 (80%)	4486 (31%)
Positive clinically only		619 (11%)	619 (4.3%)
Reason for COVID-19 test			
Symptoms	1234 (14%)	4082 (72%)	5316 (37%)
Contact tracing/risk zone travel	1703 (20%)	972 (17%)	2675 (19%)
Surveillance (healthy)	2460 (29%)	224 (4.0%)	2684 (19%)
Other/none	3230 (37%)	357 (6.3%)	3587 (25%)
Age	31.0 (27.0, 34.0)	30.0 (27.0, 34.0)	31.0 (27.0, 34.0)
Healthcare coverage	6686 (89%)	3702 (85%)	10,388 (87%)
Pre-existing condition	1112 (15%)	482 (12%)	1594 (14%)
Primiparous	3364 (46%)	1699 (42%)	5063 (44%)
Pre-pregnancy BMI (kg/m^2^)			
< 25	3218 (47%)	1782 (48%)	5000 (47%)
25–30	1775 (26%)	1023 (28%)	2798 (27%)
> = 30	1849 (27%)	902 (24%)	2751 (26%)
Continent			
Africa	378 (4.4%)	238 (4.2%)	616 (4.3%)
Asia	489 (5.7%)	413 (7.3%)	902 (6.3%)
Europe	3084 (36%)	1310 (23%)	4394 (31%)
North America	3328 (39%)	1601 (28%)	4929 (35%)
South America	1347 (16%)	2073 (37%)	3420 (24%)

^a^Descriptive characteristics of eligible participants in the International Registry of Coronavirus Exposure in Pregnancy who enrolled between June 2020 and July 2021 (*n* = 14,264)

^b^Prospective enrollment occurred during pregnancy, and retrospective enrollment during the 6 months after the end of pregnancy

COVID-19 Coronavirus disease 2019, *LMP* Last menstrual period, *BMI* Body mass index

At the time of analysis, 5884 participants had reported 5848 live births and 36 stillbirths. Of those who joined while pregnant, 4.8% had already provided outcome data, 9.3% were less than 42 weeks’ gestation at the time of this analysis or reported still being pregnant on a monthly survey, and 85.9% were more than 42 weeks but had not yet provided outcomes (i.e., presumed lost to follow-up). Of those who joined after pregnancy, 96.6% had provided at least some outcome data. Participants with positive tests or diagnoses were slightly less likely to provide outcome data or still be pregnant as those testing negative, both among prospective (12.4% of positive vs. 16.1% of negative) and retrospective pregnancies (95.0% vs. 96.9%) (Table S5 in Additional file 1).

Among the 5059 pregnant individuals with symptomatic COVID-19 (89.7% of positive participants), we classified 293 as severe, 2662 moderate, and 2104 mild (Fig. 1); 994 had available outcomes, with 30.3, 11.6, and 9.0% preterm deliveries in the severe, moderate, and mild groups, respectively (Table 2). Of preterm births in the severe group, 82.5% were C-sections, compared to 61.1% in the mild and moderate groups combined.

**Table 2:** Delivery outcomes^a^

	Negative *N* = 4692	Asymptomatic *N* = 198	Mild *N* = 389	Moderate *N* = 473	Severe *N* = 132
Preterm premature rupture of membranes	137 (2.9%)	6 (3.1%)	11 (2.8%)	17 (3.6%)	6 (4.7%)
Preterm labor	239 (5.1%)	12 (6.1%)	14 (3.6%)	40 (8.6%)	14 (11%)
Induced labor	1896 (42%)	67 (36%)	125 (34%)	177 (39%)	44 (37%)
Cesarean-section	1883 (41%)	95 (50%)	170 (45%)	220 (48%)	80 (65%)
Preterm delivery	414 (8.8%)	22 (11%)	35 (9.0%)	55 (12%)	40 (30%)
Type of delivery					
Term	4278 (91%)	176 (89%)	354 (91%)	418 (88%)	92 (70%)
Indicated preterm	132 (2.8%)	10 (5.1%)	12 (3.1%)	19 (4.0%)	17 (13%)
Spontaneous preterm	282 (6.0%)	12 (6.1%)	23 (5.9%)	36 (7.6%)	23 (17%)

^a^Delivery outcomes among eligible participants in the International Registry of Coronavirus Exposure in pregnancy who had reported outcomes by July 31, 2021 (*n* = 5884). See Additional file 1 for the survey questions defining the outcomes

### Multivariable regression

There were 5566 participants eligible for the multivariable regression analyses, of whom 876 had COVID-19 before 37 weeks’ gestation. Completed pregnancies with COVID-19 before 37 weeks were 1.3 (95% CI 1.0, 1.7) times as likely to deliver preterm as those testing negative, and those with severe disease 2.4 (1.7, 3.3) times as likely as with mild or moderate disease (Table 3). The risk ratio comparing moderate to mild disease was 1.1 (0.7, 1.7) (Table S6 in Additional file 1). Results were almost identical when we imputed missing data for the 10.8% of eligible participants missing some covariate data (Table S6 in Additional file 1).

**Table 3:** Estimates of risk of preterm birth (spontaneous and indicated, combined)^a^

	Risk ratios^b^		Standardized risks^b^			
Model	Positive vs. negative	Severe vs. mild/moderate	Negative	Positive	Mild/moderate	Severe
Log-linear regression	1.3 (1.0, 1.7)	2.4 (1.7, 3.3)				
Multinomial regression (Indicated)^c^	2.1 (1.2, 3.7)	4.6 (2.3, 9.3)				
Multinomial regression (Spontaneous)^c^	1.1 (0.7, 1.6)	2.6 (1.4, 4.8)				
Case-time-control^c^	1.2 (0.6, 2.3)	2.1 (0.4, 12.1)^d^				
Gestational-age-specific						
Week 20	1.0 (0.8, 1.2)	1.0 (0.7, 1.4)	9.8% (9.1, 10.5)	10.0% (7.8, 12.0)	10.0% (7.8, 12.0)	9.6% (6.2, 14.0)
Week 21	1.0 (0.8, 1.2)	1.0 (0.8, 1.5)	9.8% (9.1, 10.5)	9.9% (7.8, 12.0)	9.9% (7.8, 11.9)	10.2% (6.9, 14.2)
Week 22	1.0 (0.8, 1.3)	1.1 (0.8, 1.5)	9.8% (9.1, 10.5)	10.0% (7.9, 12.3)	9.9% (7.8, 12.2)	11.0% (7.5, 15.3)
Week 23	1.0 (0.8, 1.3)	1.2 (0.9, 1.6)	9.7% (9.1, 10.4)	10.2% (8.0, 12.8)	10.1% (7.9, 12.7)	12.1% (8.4, 16.7)
Week 24	1.1 (0.8, 1.4)	1.3 (1.0, 1.7)	9.7% (9.0, 10.4)	10.5% (8.3, 13.3)	10.4% (8.1, 13.0)	13.3% (9.3, 18.5)
Week 25	1.1 (0.9, 1.4)	1.4 (1.1, 1.8)	9.7% (9.0, 10.3)	10.9% (8.7, 13.5)	10.7% (8.6, 13.1)	14.8% (10.7, 19.9)
Week 26	1.2 (0.9, 1.4)	1.5 (1.2, 1.9)	9.6% (9.0, 10.3)	11.3% (9.1, 13.6)	11.0% (9.0, 13.3)	16.4% (12.1, 21.7)
Week 27	1.2 (1.0, 1.5)	1.6 (1.3, 2.0)	9.6% (8.9, 10.3)	11.7% (9.6, 13.9)	11.3% (9.2, 13.5)	18.1% (13.3, 23.6)
Week 28	1.3 (1.0, 1.5)	1.7 (1.3, 2.2)	9.5% (8.8, 10.2)	12.0% (10.0, 14.1)	11.5% (9.6, 13.5)	19.8% (14.5, 25.5)
Week 29	1.3 (1.1, 1.5)	1.9 (1.4, 2.4)	9.4% (8.8, 10.1)	12.1% (10.1, 14.1)	11.5% (9.7, 13.4)	21.4% (15.7, 27.2)
Week 30	1.3 (1.1, 1.5)	2.0 (1.5, 2.6)	9.3% (8.7, 10.0)	12.0% (10.1, 13.9)	11.4% (9.6, 13.2)	22.6% (16.7, 29.2)
Week 31	1.3 (1.1, 1.5)	2.1 (1.6, 2.8)	9.1% (8.5, 9.8)	11.7% (9.9, 13.6)	11.0% (9.2, 12.8)	23.5% (17.3, 30.4)
Week 32	1.3 (1.1, 1.5)	2.3 (1.7, 3.0)	8.9% (8.3, 9.5)	11.2% (9.4, 13.0)	10.4% (8.7, 12.1)	23.9% (17.5, 31.3)
Week 33	1.2 (1.0, 1.4)	2.5 (1.8, 3.3)	8.5% (8.0, 9.1)	10.4% (8.7, 12.0)	9.6% (8.1, 11.2)	23.6% (17.1, 31.4)
Week 34	1.2 (1.0, 1.4)	2.6 (1.9, 3.6)	7.9% (7.4, 8.4)	9.3% (7.9, 10.7)	8.5% (7.1, 9.8)	22.4% (16.0, 30.2)
Week 35	1.1 (0.9, 1.3)	2.8 (2.0, 4.0)	6.9% (6.4, 7.3)	7.6% (6.5, 8.8)	6.9% (5.9, 8.0)	19.6% (13.8, 26.6)
Week 36	1.0 (0.9, 1.2)	3.1 (2.1, 4.5)	4.8% (4.4, 5.1)	4.9% (4.2, 5.7)	4.4% (3.7, 5.1)	13.6% (9.4, 19.4)

^a^Estimates of risk of preterm birth from the various models and comparisons across levels of COVID-19. Gestational age-specific results (standardized risks and risk ratios estimated from the time-to-delivery models) are presented by week of COVID-19. Confidence intervals for the regression models were estimated with robust standard errors, and with the bootstrap for the gestational-age-specific risks and risk ratios

^b^Adjusted for continent (Africa, Asia, Europe, North America, South America), maternal age (years), pre-pregnancy BMI (kg/m^2^), parity (primi-/multiparous), race/ethnicity (Asian, Black, Latina, White, mixed, other), pre-existing condition (chronic diabetes, asthma, cardiovascular disease, or autoimmune disease), healthcare coverage (yes/no), and reason for testing (symptoms, contact tracing, surveillance, other/not tested)

^c^Odds ratios

^d^Severe vs. negative

We estimated separate odds ratios for spontaneous and indicated preterm delivery. COVID-19 during pregnancy was associated with 1.1 (0.7, 1.6) times the odds of spontaneous preterm and 2.1 (1.2, 3.7) times the odds of indicated preterm, relative to term birth. Odds ratios for severe vs. mild/moderate COVID-19 were 2.6 (1.4, 4.8) and 4.6 (2.3, 9.3) for spontaneous and indicated preterm delivery, respectively (Table 3).

### Time-to-delivery

**Fig. 2:**
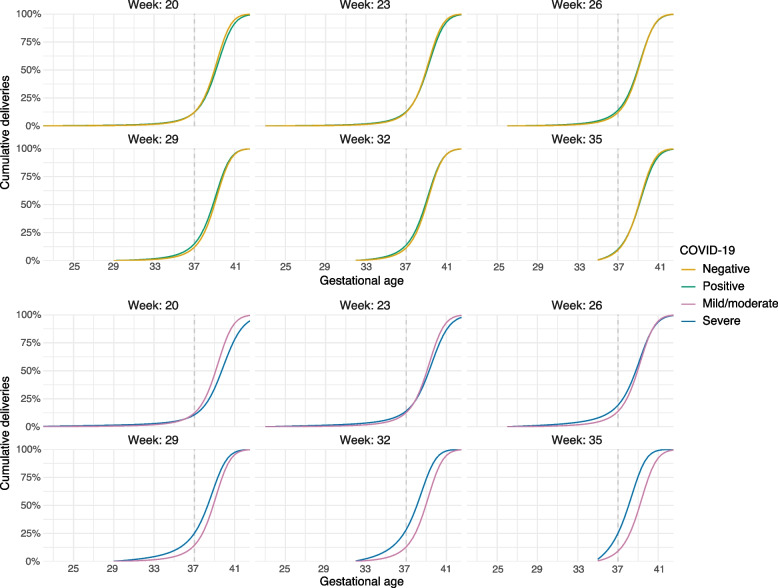
Cumulative deliveries after COVID-19 in (a selection of) weeks of pregnancy, standardized to the distribution of covariates in the test-negative population. Each panel depicts the pattern of deliveries after COVID-19 in that week; COVID-19 negative individuals in a given week are those who are still pregnant at that week. Week 20 refers to all infections at or before week 20. The risk of preterm delivery under a given condition is the percentage of deliveries that have occurred by 37 weeks, or where the curves cross the dashed line

There were 13,530 participants eligible for the time-to-delivery analyses, of whom 5053 had COVID-19 during pregnancy. Adjusted absolute risks of preterm delivery varied by gestational age (Fig. 2). For example, risk of preterm delivery after infection before 20 weeks of pregnancy was 10.0% (7.8, 12.0), and was 9.8% (9.1, 10.5) among pregnancies that were ongoing but not infected at that time, compared to 7.6% (6.5, 8.8) and 6.9% (6.4, 7.3), respectively, at 35 weeks. Risk was 9.6% (6.2, 14.0) for severe disease before 20 weeks and 19.6% (13.8, 26.6) for severe disease at 35 weeks. As with the multivariable regression, risks for mild and moderate disease were essentially identical (Table S7 in Additional file 1). Risk ratios comparing severe to mild/moderate disease at 20 and 35 weeks were 1.0 (0.7, 1.4), and 2.8 (2.0, 4.0), respectively. Table 3 contains absolute risks and risk ratios for additional weeks, and Table S4 in Additional file 1 risk differences. Results for very preterm delivery (before 34 weeks) were very similar (Table S8 in Additional file 1).

**Fig. 3:**
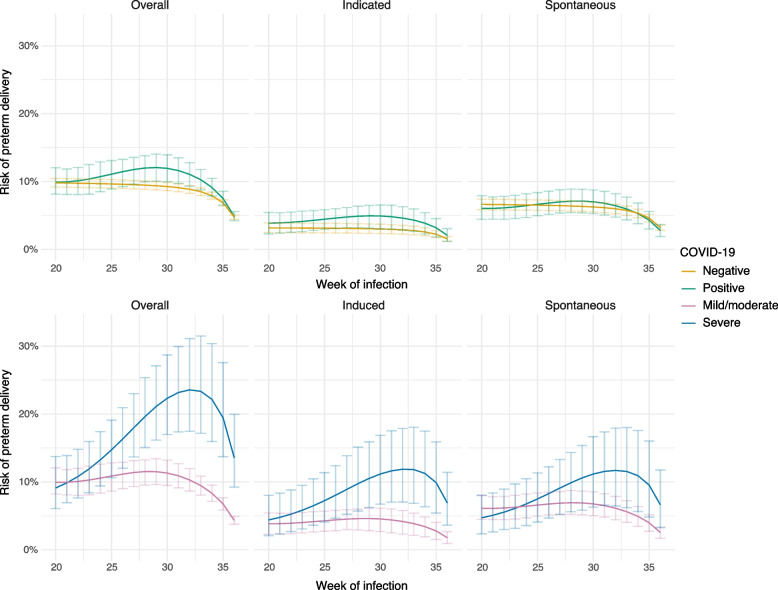
Standardized risks of preterm delivery (delivery before 37 completed weeks of gestation) after COVID-19, according to week of infection and COVID-19 severity. The risk in a given “week of infection” for the COVID-negative group is the risk for someone whose pregnancy is ongoing that week (i.e., has not yet delivered) but who doesn’t have COVID-19. The overall risk of preterm delivery (left-hand panels) has been partitioned into the risk of indicated and spontaneous preterm delivery. Confidence intervals were estimated with the bootstrap

Compared to mild/moderate disease, risks were higher for both spontaneous and indicated preterm delivery after severe COVID-19 (Fig. 3). For example, after infection at 35 weeks, risk ratios were 2.3 (1.2, 3.9) and 3.7 (2.0, 7.0) for spontaneous and indicated preterm delivery, respectively (Tables S2 and S3 in Additional file 1).

### Case-time-control

Individuals who delivered preterm had higher odds of having had COVID-19 in the month prior to their deliveries compared to 3–4 months prior (odds ratio of 1.2 (0.6, 2.3)), accounting for time trends in exposure (Table 3). In addition, they had 2.1 (0.4, 12.1) times increased odds of having had severe COVID-19 in the month prior to their preterm deliveries compared to 3–4 months prior, but only 0.7 (0.3, 1.5) times the odds of a mild or moderate infection.

## Discussion

In a large, diverse pregnancy cohort, we found that severe COVID-19 late in pregnancy may double or triple the probability of preterm delivery but that increased risk due to milder disease, or earlier in pregnancy, is likely minimal. Much of the effect of severe COVID-19, and much or all of the smaller increase with mild and moderate disease, appears due to indicated preterm deliveries. Nonetheless, severe COVID-19-affected pregnancies also reported more spontaneous preterm labour or rupture of membranes, suggesting an effect on spontaneous preterm delivery.

Intrauterine bacterial infection is a major cause of spontaneous preterm delivery [20], mediated through the innate immune response [21], and concern about the effects of SARS-CoV-2 infection during pregnancy is justified by evidence from viral infections [22, 23]. Influenza infection increases risk of preterm birth [24–27], and SARS and MERS outbreaks generated evidence of harms of coronavirus infection during pregnancy, with reports of preterm birth, intrauterine growth restriction, and mortality [28–30].

A number of meta-analyses have pooled risk of preterm birth after COVID-19 from case reports, case series, and other early studies, though estimates vary widely [31–38]. Apparent differences in risk result from selection of hospitalized patients, exclusion of ongoing pregnancies, and inclusion of pregnancies past 37 weeks’ gestation at symptom onset. Studies that include infections after 37 weeks underestimate the risk of preterm delivery, and those restricted to hospitalized patients could overestimate it or result in selection bias if factors affecting hospitalization and preterm risk are not accounted for. Our study improves upon previous estimates of risk by providing gestational-age specific risks among pregnant people with both severe and mild/moderate disease using data from ongoing (or lost to follow-up) as well as completed pregnancies.

Other studies, including some based on surveillance data, have also provided valuable comparisons with concurrent [8–12, 39–43] or historical [44–46] COVID-19-negative pregnancies, or across the spectrum of disease severity [14, 47–52]. Most [8, 10–12, 14, 43, 44, 47–52] but not all studies [40–42] have provided evidence that any vs. no infection, or more vs. less serious disease, are associated with higher preterm risk, particularly with respect to indicated deliveries. However, the extent to which they have adjusted for confounders or avoided other sources of bias differs. For example, use of historical references is questionable given the pandemic’s broad effects [53]. Moreover, studies that define exposure to COVID-19 as “any time during pregnancy” will be biased by design, given the shorter opportunity for infection in preterm deliveries, and also obscure gestational-age-specific risks.

We used three designs, each addressing shortcomings of the others, to investigate whether COVID-19 affects risk of preterm birth. We addressed confounding with multivariable adjustment as well as with within-person comparisons. In addition, in our main time-to delivery analysis, we accounted for several timing-related issues: longer pregnancies are more likely to have been exposed to SARS-CoV-2, risk of preterm delivery depends on gestational age at time zero (infection or reference), and preterm delivery cannot be assessed among participants whose pregnancies are ongoing and under 37 weeks.

Nevertheless, our study has important limitations. Information on tests and gestational age at delivery was self-reported. However, mothers who suffered COVID-19 during pregnancy are likely to remember when it occurred, and all their estimated due date and date of delivery. In addition, we have limited clinical measures compared to studies based on medical records or direct observation, which may have resulted in misclassification of spontaneous vs. indicated preterm delivery or of severity of COVID-19 cases. Furthermore, indications for delivery in people with symptomatic COVID-19 likely differed geographically and over time, limiting our ability to assess effects on spontaneous delivery. We used objective and standard measures of COVID-19 severity (e.g., ICU, ventilation, ECMO) to maximize specificity, at the possible cost of sensitivity (e.g., hospitalizations may have been precautionary due to pregnancy). When comparing to mild/moderate disease, this misclassification would tend to bias toward the null; therefore, we may have underestimated associations with severe COVID-19. While we found no differences between risks after moderate and more mild disease, it is possible that this was due to imprecision in self-reported symptoms, and future research should investigate whether there are any signs or symptoms that confer additional risk. We did not include asymptomatic participants in our main analyses so were not able to assess whether they were at higher risk than uninfected pregnancies; however, previous studies have found risk to be elevated primarily after symptomatic disease [38]. In addition, some participants who tested negative may have later developed an asymptomatic infection that was not detected or reported on a survey, so may be misclassified. Finally, participants in this study were not vaccinated, so we were not able to investigate the effect of COVID-19 vaccination.

Furthermore, while we do not have outcomes on some participants with ongoing pregnancies, many others were lost to follow-up. We sent reminders via text message to participants to encourage complete follow-up, but we were not able to increase retention [15]. In our week-specific analyses we were able to use data until the last known week of continued pregnancy under the assumption that loss to follow-up was conditionally independent of preterm birth, given the covariates measured on the initial surveys. Predictors of selection would have to be strongly associated with preterm birth to explain away the increased risk we found with severe COVID-19 [54]. In addition, although we had participants worldwide, some countries were represented more than others, and within-country sampling was not random. Because of this design, our study is not representative of any particular well-defined population, and high-risk pregnancies that are more likely to be tested may be overrepresented. While it is more widely representative than previous studies, there are also undoubtedly people who aren’t represented, in particular, those without sufficient internet access. Although the intensity of the pandemic may differ geographically, biologic effects of COVID-19 may be less likely to vary across the population. However, if the effect is mediated through precautionary early C-sections to avoid transmission or maternal complications during labour, then populations unable or hesitant to conduct these would not see an increased risk of preterm delivery; instead, risk of other periconceptional morbidities may rise.

## Conclusions

In conclusion, this study suggests that with respect to preterm birth, prevention of COVID-19 is especially important in the second half of pregnancy. However, while the risk of preterm birth appears to double or triple after severe COVID-19, the population attributable risk is likely modest, as severe COVID-19 is rare among young people, though may be more common during pregnancy [31]. Our study was not designed to estimate the risk of COVID-19 or of severe disease in pregnancy, but it is clear that protective measures should be taken to avoid SARS-CoV-2 infection and symptoms should be closely monitored to avoid progression. Much of the increased risk of prematurity due to severe disease is likely iatrogenic, due to urgent delivery in response to maternal or foetal decline. Vaccines lower the risk of infection and severe disease, and improved treatments for COVID-19 could lower risk of progression and thus prematurity by reducing indications for delivery.

## Supplementary Information

**Additional file 1:** **Table S1.** Descriptive characteristics of eligible participants in the International Registry of Coronavirus Exposure in Pregnancy who enrolled between June 2020 and July 2021 and provided outcomes by July 2021 (*n* = 5884). **Figure S1.** Raw cumulative incidence of deliveries after COVID-19 in selected weeks of gestation. COVID-19 negative individuals in a given week are those who are still pregnant at that week. Week 20 refers to all infections at or before week 20. **Figure S2.** Months since last menstrual period (LMP) at a) enrollment and b) time of symptom onset or test (if negative or positive, symptomatic), for participants who joined while pregnant and within 6 months after pregnancy, stratified by COVID-19 severity. **Table S2.** Estimates of standardized risks of spontaneous preterm delivery and risk ratios comparing COVID-19 positive vs. negative and severe vs. mild/moderate. **Table S3.** Estimates of standardized risks of induced preterm delivery and risk ratios comparing COVID-19 positive vs. negative and severe vs. mild/moderate. **Table S4.** Risk differences for overall, spontaneous, and induced preterm delivery, comparing COVID-19 positive vs. negative and severe vs. mild/moderate. **Figure S3.** Results from varying the risk window (and corresponding size of reference window) in case-time-control analysis. **Table S5.** Descriptive characteristics comparing those who provided outcome data and those who did not, but whose pregnancies were presumed completed (i.e., lost to follow-up). **Table S6.** Comparison of risk ratios from complete-case (as in the main text) and multiply-imputed analyses. **Table S7.** Estimates from various sensitivity analyses of log-linear analysis. **Figure S4.** Cumulative deliveries across gestation, estimated in various sensitivity analyses (COVID-positive vs. negative): a) Estimating risks separately for mild and moderate severity groups; b) Finer modeling of the discrete-time hazard of delivery; c) Excluding participants who joined after completion of pregnancy; d) Excluding participants who tested negative within two weeks before delivery (i.e., routine delivery testing). **Figure S5.** Cumulative deliveries across gestation, estimated in various sensitivity analyses (COVID-19 severity): a) Estimating risks separately for mild and moderate severity groups; b) Finer modeling of the discrete-time hazard of delivery; c) Excluding participants who joined after completion of pregnancy; d) Excluding participants who tested negative within two weeks before delivery (i.e., routine delivery testing). **Table S8.** Estimates of standardized risks of *very* preterm delivery (< 34 weeks) and risk ratios comparing COVID-19 positive vs. negative and severe vs. mild/moderate. **Table S9.** Estimates of standardized risks of spontaneous *very* preterm delivery (< 34 weeks) and risk ratios comparing COVID-19 positive vs. negative and severe vs. mild/moderate. **Table S10.** Estimates of standardized risks of induced *very* preterm delivery (< 34 weeks) and risk ratios comparing COVID-19 positive vs. negative and severe vs. mild/moderate. **Table S11.** Distribution of symptoms and healthcare-related actions across levels of COVID-19 severity.

## Data Availability

The data that support the findings of this study are available from Pregistry but restrictions apply to the availability of these data, which were used under license for the current study, and so are not publicly available. Data are however available from the authors upon reasonable request and with permission of Pregistry (Diego F. Wyszynski).

## Abbreviations

IRCEP: International Registry of Coronavirus Exposure in Pregnancy
COVID-19: Coronavirus disease 2019
SARS-CoV-2: Severe acute respiratory syndrome coronavirus 2
PCR: Polymerase chain reaction
ICU: Intensive care unit
ECMO: Extracorporeal membrane oxygenation
C-section: Caesarean section
LMP: Last menstrual period
BMI: Body mass index
