# Supplementary material for: Timing and severity of COVID-19 during pregnancy and risk of preterm birth in the International Registry of Coronavirus Exposure in Pregnancy
Source: BMC Pregnancy Childbirth. 2022 Oct 18;22:775. doi: 10.1186/s12884-022-05101-3 (PMC9578260; doi:10.1186/s12884-022-05101-3)
Supplement: Supplementary file 1 — Additional file 1: Table S1. Descriptive characteristics of eligible participants in the International Registry of Coronavirus Exposure in Pregnancy who enrolled between June 2020 and July 2021 and provided outcomes by July 2021 (n = 5884). Figure S1. Raw cumulative incidence of deliveries after COVID-19 in selected weeks of gestation. COVID-19 negative individuals in a given week are those who are still pregnant at that week. Week 20 refers to all infections at or before week 20. Figure S2. Months since last menstrual period (LMP) at a) enrollment and b) time of symptom onset or test (if negative or positive, symptomatic), for participants who joined while pregnant and within 6 months after pregnancy, stratified by COVID-19 severity. Table S2. Estimates of standardized risks of spontaneous preterm delivery and risk ratios comparing COVID-19 positive vs. negative and severe vs. mild/moderate. Table S3. Estimates of standardized risks of induced preterm delivery and risk ratios comparing COVID-19 positive vs. negative and severe vs. mild/moderate. Table S4. Risk differences for overall, spontaneous, and induced preterm delivery, comparing COVID-19 positive vs. negative and severe vs. mild/moderate. Figure S3. Results from varying the risk window (and corresponding size of reference window) in case-time-control analysis. Table S5. Descriptive characteristics comparing those who provided outcome data and those who did not, but whose pregnancies were presumed completed (i.e., lost to follow-up). Table S6. Comparison of risk ratios from complete-case (as in the main text) and multiply-imputed analyses. Table S7. Estimates from various sensitivity analyses of log-linear analysis. Figure S4. Cumulative deliveries across gestation, estimated in various sensitivity analyses (COVID-positive vs. negative): a) Estimating risks separately for mild and moderate severity groups; b) Finer modeling of the discrete-time hazard of delivery; c) Excluding participants who joined after c [file 12884_2022_5101_MOESM1_ESM.docx]

# Supplementary Material

## Sensitivity analyses

Estimates from the log-linear analyses were almost identical when we imputed baseline covariates, when we adjusted for loss to follow-up using inverse probability of censoring weights, as well as when we excluded participants who had only a clinical diagnosis of COVID-19 and no positive test (Table S6). Varying the cutoff for time since last menstrual period in the log-linear analyses also resulted in almost identical estimates (Table S7), as did excluding participants whose negative tests were in the final two weeks of delivery (Figure S4). Estimating risks separately for mild and moderate disease had almost identical results as combining the groups (Table S6, Figure S5). Restricting our sample to North American participants only resulted in similar risk ratios (Table S7). When we restricted the analysis to prospective participants only, there was a smaller difference in risk between severe and mild/moderate disease (Figure S5), likely because of the relatively small number of individuals joining after severe disease but before delivery late in pregnancy. Varying timing and duration of the risk period in the case-time control analysis confirmed our hypothesis that risk due to infection occurred in the month prior to delivery, with particularly strong risk in the 2 weeks preceding delivery (Figure S3).

## Key survey questions used to determine outcomes

| **Outcome** | **Survey question** | **Response** |
| --- | --- | --- |
| Preterm premature rupture of membranes | Please select the pregnancy complications you had, if any (choose all that apply). | Water broke before 37 weeks |
| Preterm labor | Please select the pregnancy complications you had, if any (choose all that apply). | Preterm labor (labor before 37 weeks gestation) |
| Induced labor | Was labor induced? | Yes |
| Cesarean section | How did you deliver your baby? | Cesarean section (C-section) *(vs. vaginally)* |
| Gestational age at delivery | How many weeks pregnant were you when you delivered the baby?  Do you know your estimated due date based on your last menstrual period?  When was your due date based on the ultrasound?  On what date did you deliver? |  |

## Supplementary Tables and Figures

Table S1: Descriptive characteristics of eligible participants in the International Registry of Coronavirus Exposure in Pregnancy who enrolled between June 2020 and July 2021 and provided outcomes by July 2021 (n = 5884).

|  | COVID-19 negative N = 4692 | COVID-19 positive N = 1192 | Total N = 5884 |
| --- | --- | --- | --- |
| Enrollment |  |  |  |
| Prospective | 243 (5.2%) | 167 (14%) | 410 (7.0%) |
| Retrospective | 4,449 (95%) | 1,025 (86%) | 5,474 (93%) |
| Prospective enrolees |  |  |  |
| Weeks past LMP at enrollment (median (IQR)) | 34 (31, 38) | 33 (29, 37) | 34 (30, 37) |
| Weeks past LMP at symptom onset/test (median (IQR)) | 28 (22, 34) | 24 (16, 31) | 27 (20, 33) |
| Retrospective enrolees |  |  |  |
| Weeks past LMP at enrollment (median (IQR)) | 49 (44, 54) | 47 (43, 53) | 49 (44, 54) |
| Weeks past LMP at symptom onset/test (median (IQR)) | 38 (37, 40) | 34 (28, 38) | 38 (36, 39) |
| COVID-19 severity |  |  |  |
| Negative | 4,692 (100%) |  | 4,692 (80%) |
| Asymptomatic |  | 198 (17%) | 198 (3.4%) |
| Mild |  | 389 (33%) | 389 (6.6%) |
| Moderate |  | 473 (40%) | 473 (8.0%) |
| Severe |  | 132 (11%) | 132 (2.2%) |
| COVID-19 diagnosis/test type |  |  |  |
| Negative | 4,692 (100%) |  | 4,692 (80%) |
| Positive by antibodies only |  | 151 (13%) | 151 (2.6%) |
| Positive by throat/nose swab |  | 881 (74%) | 881 (15%) |
| Positive clinically only |  | 160 (13%) | 160 (2.7%) |
| Reason for COVID-19 test |  |  |  |
| Symptoms | 297 (6.3%) | 732 (61%) | 1,029 (17%) |
| Contact tracing/risk zone travel | 448 (9.5%) | 194 (16%) | 642 (11%) |
| Surveillance (healthy) | 1,609 (34%) | 91 (7.6%) | 1,700 (29%) |
| Other/none | 2,338 (50%) | 175 (15%) | 2,513 (43%) |
| Age | 31.0 (27.0, 34.0) | 30.0 (27.0, 34.0) | 31.0 (27.0, 34.0) |
| Healthcare coverage | 4,081 (90%) | 989 (86%) | 5,070 (89%) |
| Pre-existing condition | 664 (15%) | 121 (11%) | 785 (14%) |
| Primiparous | 2,137 (47%) | 468 (42%) | 2,605 (46%) |
| Pre-pregnancy BMI |  |  |  |
| <25 | 1,943 (45%) | 525 (48%) | 2,468 (46%) |
| 25-30 | 1,117 (26%) | 300 (27%) | 1,417 (26%) |
| >= 30 | 1,239 (29%) | 270 (25%) | 1,509 (28%) |
| Continent |  |  |  |
| Africa | 228 (4.9%) | 63 (5.3%) | 291 (4.9%) |
| Asia | 232 (4.9%) | 69 (5.8%) | 301 (5.1%) |
| Europe | 1,711 (36%) | 311 (26%) | 2,022 (34%) |
| North America | 2,016 (43%) | 423 (36%) | 2,439 (41%) |
| South America | 504 (11%) | 325 (27%) | 829 (14%) |


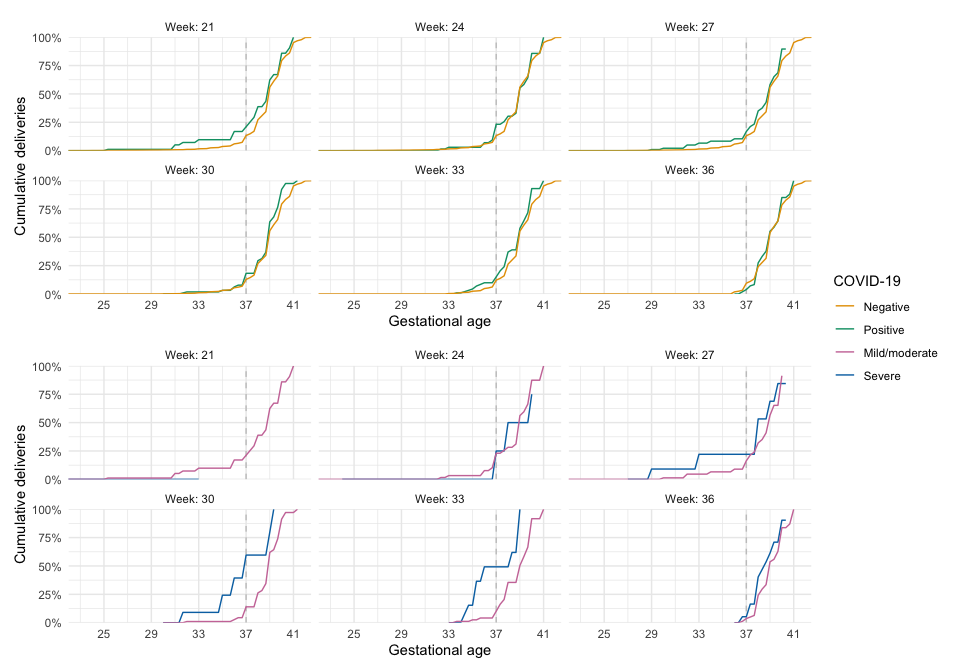


Figure S1: Raw cumulative incidence of deliveries after COVID-19 in selected weeks of gestation. COVID-19 negative individuals in a given week are those who are still pregnant at that week. Week 20 refers to all infections at or before week 20.


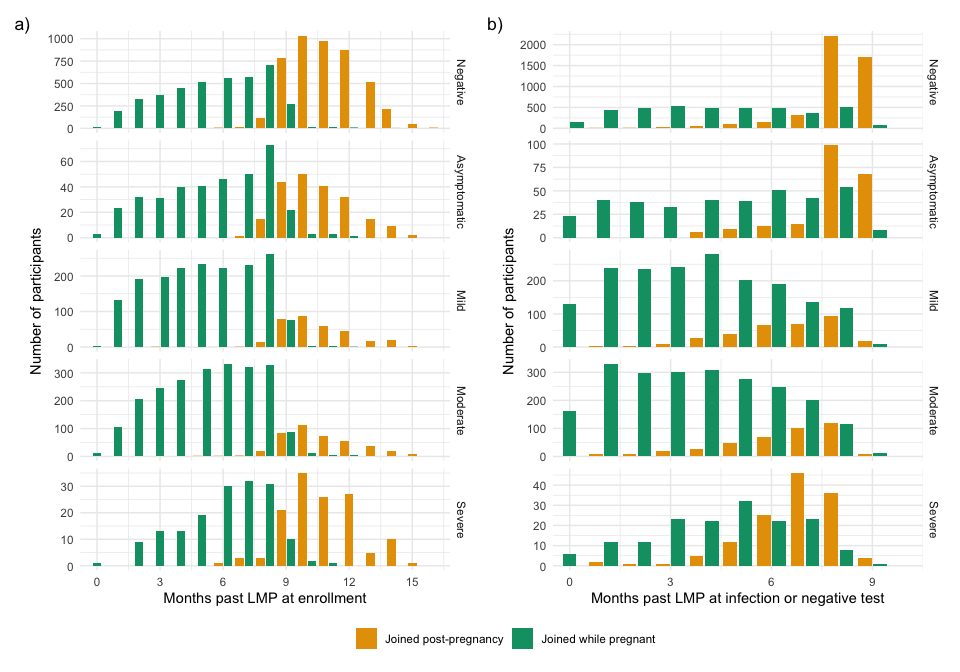


Figure S2: Months since last menstrual period (LMP) at a) enrollment and b) time of symptom onset or test (if negative or positive, symptomatic), for participants who joined while pregnant and within 6 months after pregnancy, stratified by COVID-19 severity.

Table S2: Estimates of standardized risks of spontaneous preterm delivery and risk ratios comparing COVID-19 positive vs. negative and severe vs. mild/moderate.

|  | Negative | Positive | Positive vs. negative | Mild/moderate | Severe | Severe vs. mild/moderate |
| --- | --- | --- | --- | --- | --- | --- |
| Week 20 | 6.6% (5.9, 7.5) | 6.1% (4.4, 8.0) | 0.9 (0.7, 1.2) | 6.2% (4.5, 8.1) | 4.8% (2.4, 8.3) | 0.8 (0.4, 1.2) |
| Week 21 | 6.6% (5.8, 7.4) | 6.1% (4.4, 7.9) | 0.9 (0.7, 1.2) | 6.1% (4.5, 7.9) | 5.1% (2.5, 8.7) | 0.8 (0.4, 1.3) |
| Week 22 | 6.6% (5.8, 7.4) | 6.1% (4.4, 8.1) | 0.9 (0.7, 1.2) | 6.1% (4.4, 8.1) | 5.5% (2.7, 9.3) | 0.9 (0.5, 1.4) |
| Week 23 | 6.6% (5.8, 7.4) | 6.2% (4.4, 8.1) | 0.9 (0.7, 1.3) | 6.2% (4.4, 8.2) | 6.0% (3.0, 10.3) | 1.0 (0.5, 1.5) |
| Week 24 | 6.6% (5.8, 7.4) | 6.4% (4.5, 8.3) | 1.0 (0.7, 1.3) | 6.3% (4.5, 8.3) | 6.6% (3.4, 11.2) | 1.0 (0.6, 1.6) |
| Week 25 | 6.5% (5.8, 7.3) | 6.6% (4.7, 8.5) | 1.0 (0.7, 1.3) | 6.5% (4.7, 8.4) | 7.3% (3.9, 11.9) | 1.1 (0.6, 1.7) |
| Week 26 | 6.5% (5.7, 7.3) | 6.8% (5.0, 8.6) | 1.0 (0.8, 1.4) | 6.7% (4.9, 8.6) | 8.1% (4.3, 12.6) | 1.2 (0.7, 1.8) |
| Week 27 | 6.5% (5.7, 7.3) | 7.0% (5.2, 8.8) | 1.1 (0.8, 1.4) | 6.8% (5.1, 8.8) | 8.9% (4.7, 13.3) | 1.3 (0.7, 2.0) |
| Week 28 | 6.4% (5.6, 7.2) | 7.1% (5.4, 8.9) | 1.1 (0.8, 1.4) | 6.9% (5.2, 8.9) | 9.7% (5.2, 14.2) | 1.4 (0.8, 2.1) |
| Week 29 | 6.3% (5.6, 7.1) | 7.1% (5.5, 9.1) | 1.1 (0.9, 1.4) | 6.9% (5.2, 9.0) | 10.4% (5.8, 15.5) | 1.5 (0.8, 2.3) |
| Week 30 | 6.3% (5.5, 7.0) | 7.1% (5.3, 9.2) | 1.1 (0.9, 1.5) | 6.8% (5.1, 8.9) | 10.9% (6.0, 16.6) | 1.6 (0.9, 2.5) |
| Week 31 | 6.2% (5.4, 6.9) | 6.9% (5.2, 8.8) | 1.1 (0.8, 1.4) | 6.6% (4.9, 8.5) | 11.3% (6.2, 17.2) | 1.7 (1.0, 2.7) |
| Week 32 | 6.0% (5.3, 6.7) | 6.5% (4.9, 8.3) | 1.1 (0.8, 1.4) | 6.2% (4.5, 8.0) | 11.4% (6.2, 17.6) | 1.8 (1.0, 3.0) |
| Week 33 | 5.7% (5.1, 6.4) | 6.0% (4.4, 7.7) | 1.0 (0.8, 1.4) | 5.7% (4.1, 7.4) | 11.2% (6.0, 17.5) | 2.0 (1.1, 3.3) |
| Week 34 | 5.3% (4.7, 6.0) | 5.3% (3.9, 6.8) | 1.0 (0.7, 1.3) | 5.0% (3.5, 6.5) | 10.6% (5.6, 16.9) | 2.1 (1.1, 3.6) |
| Week 35 | 4.6% (4.1, 5.2) | 4.3% (3.1, 5.7) | 0.9 (0.7, 1.2) | 4.0% (2.8, 5.4) | 9.2% (4.9, 15.4) | 2.3 (1.2, 3.9) |
| Week 36 | 3.2% (2.8, 3.6) | 2.8% (2.0, 3.7) | 0.9 (0.6, 1.2) | 2.6% (1.8, 3.5) | 6.4% (3.3, 10.9) | 2.5 (1.3, 4.3) |

Table S3: Estimates of standardized risks of induced preterm delivery and risk ratios comparing COVID-19 positive vs. negative and severe vs. mild/moderate.

|  | Negative | Positive | Positive vs. negative | Mild/moderate | Severe | Severe vs. mild/moderate |
| --- | --- | --- | --- | --- | --- | --- |
| Week 20 | 3.2% (2.5, 3.9) | 3.8% (2.4, 5.7) | 1.2 (0.7, 1.8) | 3.8% (2.3, 5.7) | 4.7% (2.1, 8.5) | 1.2 (0.7, 2.2) |
| Week 21 | 3.2% (2.5, 3.9) | 3.8% (2.4, 5.4) | 1.2 (0.8, 1.8) | 3.8% (2.3, 5.3) | 5.0% (2.4, 9.0) | 1.3 (0.7, 2.4) |
| Week 22 | 3.2% (2.5, 3.9) | 3.9% (2.4, 5.5) | 1.2 (0.8, 1.8) | 3.8% (2.3, 5.4) | 5.5% (2.8, 9.5) | 1.4 (0.8, 2.5) |
| Week 23 | 3.2% (2.5, 3.9) | 4.0% (2.5, 5.6) | 1.3 (0.8, 1.8) | 3.9% (2.4, 5.5) | 6.0% (3.2, 10.2) | 1.6 (0.9, 2.7) |
| Week 24 | 3.1% (2.5, 3.9) | 4.2% (2.7, 5.8) | 1.3 (0.9, 1.9) | 4.0% (2.6, 5.7) | 6.7% (3.6, 11.1) | 1.7 (0.9, 3.0) |
| Week 25 | 3.1% (2.5, 3.9) | 4.3% (2.9, 6.0) | 1.4 (0.9, 1.9) | 4.2% (2.8, 5.8) | 7.5% (4.1, 12.1) | 1.8 (1.0, 3.2) |
| Week 26 | 3.1% (2.5, 3.8) | 4.5% (3.2, 6.0) | 1.5 (1.0, 2.0) | 4.3% (2.9, 5.8) | 8.3% (4.7, 13.2) | 1.9 (1.1, 3.4) |
| Week 27 | 3.1% (2.5, 3.8) | 4.7% (3.3, 6.3) | 1.5 (1.1, 2.1) | 4.5% (3.0, 5.9) | 9.2% (5.4, 14.4) | 2.1 (1.2, 3.7) |
| Week 28 | 3.1% (2.5, 3.8) | 4.9% (3.4, 6.3) | 1.6 (1.1, 2.1) | 4.6% (3.0, 5.9) | 10.2% (6.0, 15.6) | 2.2 (1.3, 4.0) |
| Week 29 | 3.1% (2.5, 3.8) | 5.0% (3.4, 6.5) | 1.6 (1.2, 2.2) | 4.6% (3.0, 6.1) | 11.0% (6.5, 16.7) | 2.4 (1.4, 4.2) |
| Week 30 | 3.0% (2.4, 3.7) | 5.0% (3.3, 6.6) | 1.7 (1.1, 2.2) | 4.6% (2.9, 6.0) | 11.7% (7.0, 17.8) | 2.6 (1.5, 4.5) |
| Week 31 | 3.0% (2.4, 3.6) | 4.9% (3.2, 6.5) | 1.7 (1.1, 2.3) | 4.4% (2.7, 5.9) | 12.2% (7.2, 18.6) | 2.7 (1.6, 4.9) |
| Week 32 | 2.9% (2.3, 3.5) | 4.7% (3.0, 6.3) | 1.6 (1.1, 2.3) | 4.2% (2.5, 5.7) | 12.5% (7.3, 19.1) | 2.9 (1.7, 5.4) |
| Week 33 | 2.8% (2.2, 3.4) | 4.4% (2.7, 5.9) | 1.6 (1.0, 2.2) | 3.9% (2.3, 5.4) | 12.4% (7.1, 19.0) | 3.2 (1.8, 5.8) |
| Week 34 | 2.6% (2.1, 3.2) | 4.0% (2.4, 5.4) | 1.5 (1.0, 2.2) | 3.5% (1.9, 4.9) | 11.8% (6.7, 18.2) | 3.4 (1.9, 6.3) |
| Week 35 | 2.2% (1.8, 2.7) | 3.3% (1.9, 4.6) | 1.5 (0.9, 2.1) | 2.8% (1.5, 4.1) | 10.4% (5.9, 16.1) | 3.7 (2.0, 7.0) |
| Week 36 | 1.5% (1.2, 1.9) | 2.1% (1.2, 3.0) | 1.4 (0.8, 2.0) | 1.8% (1.0, 2.7) | 7.2% (4.2, 11.8) | 4.0 (2.2, 7.7) |

Table S4: Risk differences for overall, spontaneous, and induced preterm delivery, comparing COVID-19 positive vs. negative and severe vs. mild/moderate.

|  | Positive vs. negative | Severe vs. mild/moderate | Positive vs. negative  (Spontaneous) | Severe vs. mild/moderate  (Spontaneous) | Positive vs. negative  (Induced) | Severe vs. mild/moderate  (Induced) |
| --- | --- | --- | --- | --- | --- | --- |
| Week 20 | 0.2% (-2.3, 2.2) | -0.5% (-3.4, 3.6) | -0.5% (-2.4, 1.5) | -1.4% (-4.2, 1.6) | 0.7% (-0.9, 2.4) | 0.9% (-1.2, 4.2) |
| Week 21 | 0.1% (-2.1, 2.1) | 0.3% (-2.6, 4.2) | -0.6% (-2.4, 1.3) | -1.0% (-3.8, 2.0) | 0.7% (-0.8, 2.3) | 1.3% (-1.0, 4.7) |
| Week 22 | 0.2% (-2.0, 2.6) | 1.1% (-1.8, 4.9) | -0.5% (-2.3, 1.4) | -0.6% (-3.5, 2.5) | 0.7% (-0.7, 2.4) | 1.7% (-0.8, 5.3) |
| Week 23 | 0.5% (-1.9, 3.1) | 2.0% (-0.9, 6.1) | -0.4% (-2.3, 1.7) | -0.2% (-3.2, 3.1) | 0.9% (-0.6, 2.5) | 2.1% (-0.5, 6.0) |
| Week 24 | 0.8% (-1.5, 3.6) | 3.0% (0.0, 7.2) | -0.2% (-2.1, 1.9) | 0.3% (-2.9, 3.8) | 1.0% (-0.4, 2.7) | 2.7% (-0.2, 6.8) |
| Week 25 | 1.3% (-1.0, 3.9) | 4.1% (1.0, 8.5) | 0.0% (-1.9, 2.1) | 0.8% (-2.6, 4.5) | 1.2% (-0.2, 2.8) | 3.3% (0.2, 7.7) |
| Week 26 | 1.7% (-0.5, 4.2) | 5.4% (1.9, 10.2) | 0.3% (-1.6, 2.3) | 1.4% (-2.3, 5.3) | 1.4% (0.0, 2.9) | 4.0% (0.6, 8.7) |
| Week 27 | 2.1% (0.1, 4.5) | 6.8% (3.1, 11.8) | 0.5% (-1.3, 2.4) | 2.1% (-2.0, 6.0) | 1.6% (0.2, 3.1) | 4.8% (1.0, 10.0) |
| Week 28 | 2.5% (0.4, 4.6) | 8.3% (4.0, 13.5) | 0.7% (-1.0, 2.6) | 2.7% (-1.6, 7.1) | 1.8% (0.4, 3.2) | 5.6% (1.5, 11.5) |
| Week 29 | 2.7% (0.8, 4.7) | 9.8% (5.0, 15.6) | 0.8% (-0.9, 2.7) | 3.4% (-1.1, 8.2) | 1.9% (0.5, 3.4) | 6.4% (1.9, 12.8) |
| Week 30 | 2.8% (0.8, 4.7) | 11.2% (6.0, 17.2) | 0.8% (-0.9, 2.8) | 4.1% (-0.7, 9.2) | 2.0% (0.4, 3.5) | 7.1% (2.3, 13.8) |
| Week 31 | 2.6% (0.7, 4.5) | 12.5% (6.8, 18.8) | 0.7% (-1.0, 2.6) | 4.7% (-0.3, 10.1) | 1.9% (0.3, 3.5) | 7.8% (2.8, 14.6) |
| Week 32 | 2.3% (0.5, 4.1) | 13.4% (7.3, 20.3) | 0.5% (-1.2, 2.4) | 5.2% (0.2, 10.9) | 1.8% (0.2, 3.3) | 8.2% (3.2, 15.3) |
| Week 33 | 1.9% (0.2, 3.5) | 14.0% (7.5, 21.3) | 0.3% (-1.4, 2.1) | 5.5% (0.5, 11.4) | 1.6% (0.1, 3.2) | 8.5% (3.5, 15.1) |
| Week 34 | 1.4% (-0.1, 2.8) | 13.9% (7.4, 21.4) | 0.0% (-1.5, 1.6) | 5.6% (0.7, 11.7) | 1.4% (-0.1, 2.8) | 8.3% (3.6, 14.5) |
| Week 35 | 0.7% (-0.4, 1.9) | 12.7% (6.8, 19.8) | -0.3% (-1.5, 1.1) | 5.2% (0.9, 11.3) | 1.0% (-0.2, 2.3) | 7.5% (3.3, 13.3) |
| Week 36 | 0.2% (-0.6, 1.0) | 9.2% (4.9, 14.8) | -0.4% (-1.3, 0.5) | 3.8% (0.8, 8.6) | 0.6% (-0.3, 1.5) | 5.4% (2.3, 9.6) |


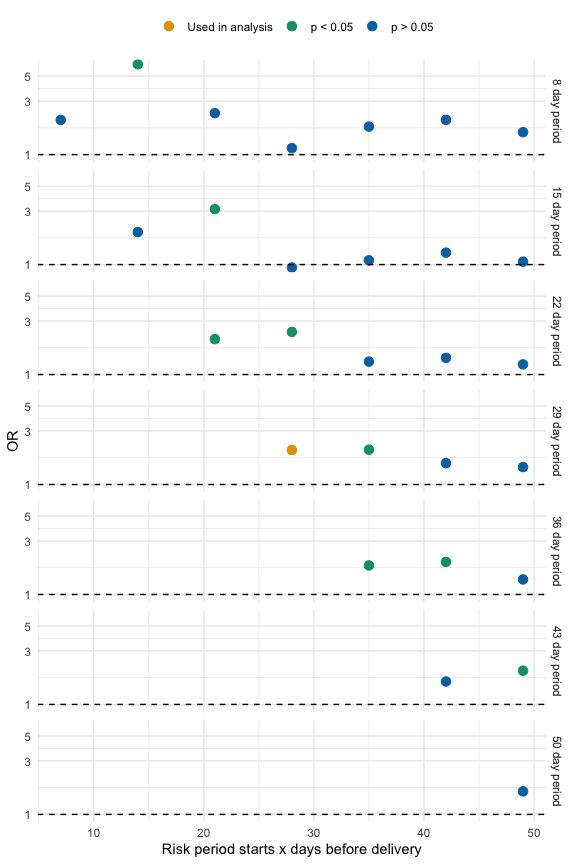


Figure S3: Results from varying the risk window (and corresponding size of reference window) in case-time-control analysis

Table S5: Descriptive characteristics comparing those who provided outcome data and those who did not, but whose pregnancies were presumed completed (i.e., lost to follow-up).

|  | No outcome data N = 7577 | Provided outcome data N = 5884 | Total N = 13461 |
| --- | --- | --- | --- |
| Enrollment |  |  |  |
| Prospective | 7,375 (97%) | 410 (7.0%) | 7,785 (58%) |
| Retrospective | 202 (2.7%) | 5,474 (93%) | 5,676 (42%) |
| Prospective enrolees |  |  |  |
| Weeks past LMP at enrollment (median (IQR)) | 27 (18, 34) | 34 (30, 37) | 27 (19, 34) |
| Weeks past LMP at symptom onset/test (median (IQR)) | 20 (12, 29) | 27 (20, 33) | 21 (13, 29) |
| Retrospective enrolees |  |  |  |
| Weeks past LMP at enrollment (median (IQR)) | 46 (41, 53) | 49 (44, 54) | 49 (44, 54) |
| Weeks past LMP at symptom onset/test (median (IQR)) | 37 (28, 39) | 38 (36, 39) | 38 (35, 39) |
| Weeks past LMP at loss to follow-up (median (IQR)) | 27 (19, 35) |  | 27 (19, 35) |
| COVID-19 severity |  |  |  |
| Asymptomatic | 352 (8.7%) | 198 (17%) | 550 (11%) |
| Mild | 1,534 (38%) | 389 (33%) | 1,923 (37%) |
| Moderate | 2,007 (50%) | 473 (40%) | 2,480 (47%) |
| Severe | 151 (3.7%) | 132 (11%) | 283 (5.4%) |
| COVID-19 diagnosis/test type |  |  |  |
| Negative | 3,533 (47%) | 4,692 (80%) | 8,225 (61%) |
| Positive by antibodies only | 349 (4.6%) | 151 (2.6%) | 500 (3.7%) |
| Positive by throat/nose swab | 3,276 (43%) | 881 (15%) | 4,157 (31%) |
| Positive clinically only | 419 (5.5%) | 160 (2.7%) | 579 (4.3%) |
| Reason for COVID-19 test |  |  |  |
| Symptoms | 3,873 (51%) | 1,029 (17%) | 4,902 (36%) |
| Contact tracing/risk zone travel | 1,840 (24%) | 642 (11%) | 2,482 (18%) |
| Surveillance (healthy) | 873 (12%) | 1,700 (29%) | 2,573 (19%) |
| Other/none | 989 (13%) | 2,513 (43%) | 3,502 (26%) |
| COVID-19 positive, prospective | 3,988 (54%) | 167 (41%) | 4,155 (53%) |
| COVID-19 positive, retrospective | 56 (28%) | 1,025 (19%) | 1,081 (19%) |
| Age | 30.0 (27.0, 34.0) | 31.0 (27.0, 34.0) | 31.0 (27.0, 34.0) |
| Healthcare coverage | 4,710 (85%) | 5,070 (89%) | 9,780 (87%) |
| Pre-existing condition | 707 (14%) | 785 (14%) | 1,492 (14%) |
| Primiparous | 2,182 (43%) | 2,605 (46%) | 4,787 (45%) |
| Pre-pregnancy BMI |  |  |  |
| <25 | 2,214 (49%) | 2,468 (46%) | 4,682 (47%) |
| 25-30 | 1,210 (27%) | 1,417 (26%) | 2,627 (26%) |
| >= 30 | 1,108 (24%) | 1,509 (28%) | 2,617 (26%) |
| Continent |  |  |  |
| Africa | 280 (3.7%) | 291 (4.9%) | 571 (4.2%) |
| Asia | 572 (7.5%) | 301 (5.1%) | 873 (6.5%) |
| Europe | 2,085 (28%) | 2,022 (34%) | 4,107 (31%) |
| North America | 2,221 (29%) | 2,439 (41%) | 4,660 (35%) |
| South America | 2,419 (32%) | 829 (14%) | 3,248 (24%) |

Table S6: Comparison of risk ratios from complete-case (as in the main text) and multiply-imputed analyses.

| Analysis | Positive vs. negative | Moderate vs. mild | Severe vs. mild |
| --- | --- | --- | --- |
| Complete-case | 1.3 (1.0, 1.7) | 1.1 (0.7, 1.7) | 2.5 (1.6, 3.9) |
| Multiply-imputed | 1.3 (1.0, 1.7) | 1.2 (0.8, 1.2) | 2.7 (1.8, 4.2) |

Table S7: Estimates from various sensitivity analyses of log-linear analysis

| Analysis | Positive vs. negative | Severe vs. mild/moderate | Moderate vs. mild | Severe vs. mild |
| --- | --- | --- | --- | --- |
| Original | 1.3 (1.0, 1.7) | 2.4 (1.7, 3.3) |  |  |
| Separate mild and moderate |  |  | 1.1 (0.7, 1.7) | 2.5 (1.6, 3.9) |
| Exclude clinical | 1.4 (1.1, 1.9) | 2.3 (1.6, 3.3) |  |  |
| North America | 0.8 (0.5, 1.3) | 2.1 (1.0, 4.4) |  |  |
| Exclude negative w/in 2 weeks | 1.1 (0.7, 1.7) |  |  |  |
| Shorter cutoff | 1.3 (1.0, 1.7) | 2.4 (1.7, 3.3) |  |  |
| Longer cutoff | 1.3 (1.0, 1.7) | 2.4 (1.7, 3.3) |  |  |
| Very preterm | 1.5 (0.8, 2.8) | 2.9 (1.6, 5.3) |  |  |
| Inverse-probability weighted | 1.2 (0.9, 1.7) | 2.5 (1.8, 3.5) |  |  |


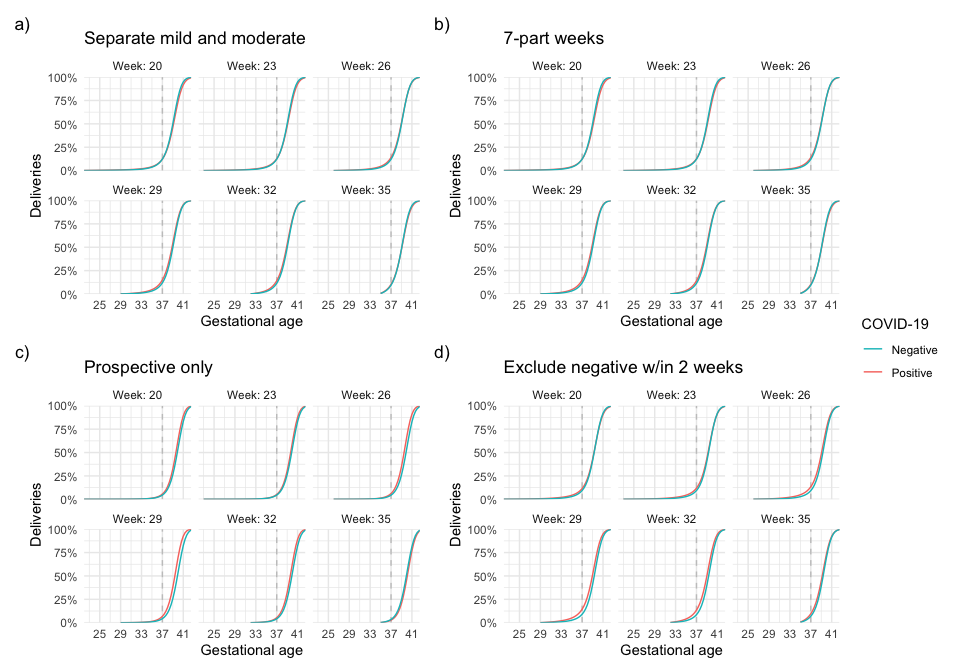


Figure S4: Cumulative deliveries across gestation, estimated in various sensitivity analyses (COVID-positive vs. negative): a) Estimating risks separately for mild and moderate severity groups; b) Finer modeling of the discrete-time hazard of delivery; c) Excluding participants who joined after completion of pregnancy; d) Excluding participants who tested negative within two weeks before delivery (i.e., routine delivery testing).


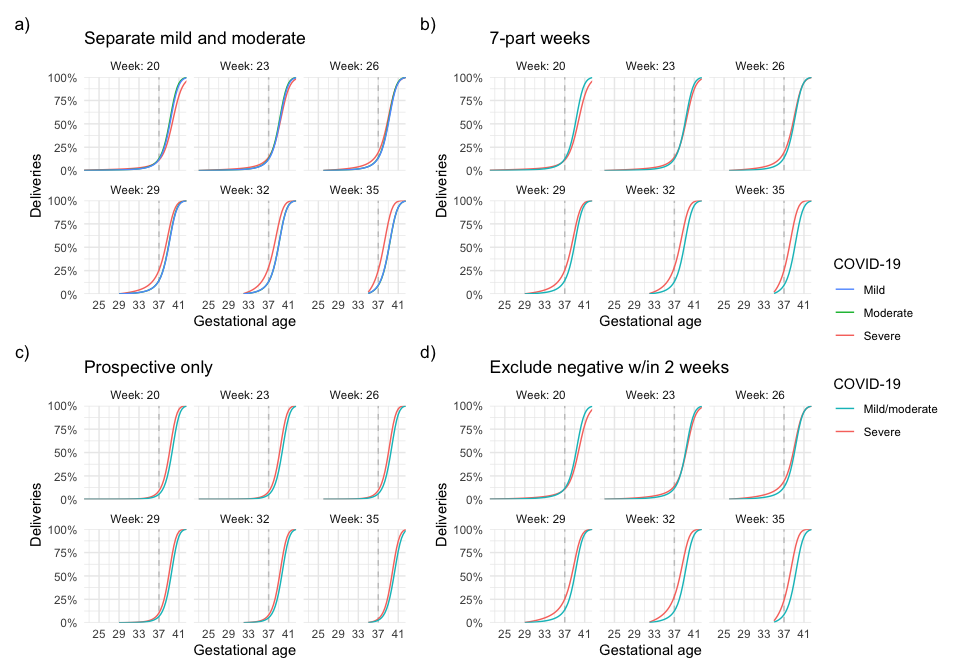


Figure S5: Cumulative deliveries across gestation, estimated in various sensitivity analyses (COVID-19 severity): a) Estimating risks separately for mild and moderate severity groups; b) Finer modeling of the discrete-time hazard of delivery; c) Excluding participants who joined after completion of pregnancy; d) Excluding participants who tested negative within two weeks before delivery (i.e., routine delivery testing).

Table S8: Estimates of standardized risks of *very* preterm delivery (< 34 weeks) and risk ratios comparing COVID-19 positive vs. negative and severe vs. mild/moderate.

|  | Negative | Positive | Positive vs. negative | Mild/moderate | Severe | Severe vs. mild/moderate |
| --- | --- | --- | --- | --- | --- | --- |
| Week 20 | 2.0% (1.7, 2.3) | 2.5% (1.7, 3.3) | 1.2 (0.8, 1.7) | 2.4% (1.6, 3.2) | 3.5% (2.1, 5.3) | 1.4 (1.1, 1.9) |
| Week 21 | 2.0% (1.7, 2.3) | 2.4% (1.7, 3.1) | 1.2 (0.8, 1.6) | 2.4% (1.6, 3.0) | 3.6% (2.2, 5.4) | 1.5 (1.1, 2.0) |
| Week 22 | 2.0% (1.6, 2.3) | 2.4% (1.6, 3.2) | 1.2 (0.8, 1.7) | 2.3% (1.6, 3.1) | 3.8% (2.3, 5.7) | 1.6 (1.2, 2.1) |
| Week 23 | 1.9% (1.6, 2.3) | 2.4% (1.6, 3.2) | 1.3 (0.9, 1.7) | 2.3% (1.6, 3.1) | 4.0% (2.5, 6.1) | 1.7 (1.3, 2.3) |
| Week 24 | 1.9% (1.6, 2.2) | 2.5% (1.7, 3.3) | 1.3 (0.9, 1.8) | 2.3% (1.6, 3.1) | 4.3% (2.7, 6.5) | 1.8 (1.4, 2.4) |
| Week 25 | 1.8% (1.6, 2.2) | 2.5% (1.7, 3.3) | 1.4 (0.9, 1.8) | 2.4% (1.6, 3.1) | 4.6% (2.9, 7.0) | 2.0 (1.5, 2.6) |
| Week 26 | 1.8% (1.5, 2.1) | 2.5% (1.8, 3.3) | 1.4 (1.0, 1.9) | 2.4% (1.7, 3.1) | 5.0% (3.1, 7.3) | 2.1 (1.6, 2.8) |
| Week 27 | 1.7% (1.5, 2.0) | 2.5% (1.8, 3.3) | 1.5 (1.1, 1.9) | 2.4% (1.7, 3.0) | 5.3% (3.4, 7.7) | 2.2 (1.6, 3.0) |
| Week 28 | 1.7% (1.4, 2.0) | 2.5% (1.8, 3.2) | 1.5 (1.1, 1.9) | 2.3% (1.7, 2.9) | 5.5% (3.5, 8.2) | 2.4 (1.7, 3.2) |
| Week 29 | 1.6% (1.3, 1.8) | 2.4% (1.8, 3.0) | 1.5 (1.1, 1.9) | 2.2% (1.6, 2.8) | 5.6% (3.6, 8.2) | 2.6 (1.8, 3.5) |
| Week 30 | 1.4% (1.2, 1.7) | 2.2% (1.6, 2.8) | 1.5 (1.2, 1.9) | 2.0% (1.5, 2.5) | 5.5% (3.5, 8.1) | 2.7 (1.9, 3.8) |
| Week 31 | 1.3% (1.1, 1.5) | 1.9% (1.4, 2.4) | 1.5 (1.1, 1.9) | 1.7% (1.3, 2.2) | 5.0% (3.2, 7.6) | 2.9 (2.0, 4.2) |
| Week 32 | 1.0% (0.9, 1.2) | 1.5% (1.1, 1.9) | 1.5 (1.1, 1.8) | 1.3% (1.0, 1.7) | 4.2% (2.6, 6.4) | 3.1 (2.1, 4.6) |
| Week 33 | 0.6% (0.6, 0.7) | 0.9% (0.7, 1.1) | 1.4 (1.1, 1.7) | 0.8% (0.6, 1.0) | 2.6% (1.6, 4.0) | 3.3 (2.2, 5.0) |

Table S9: Estimates of standardized risks of spontaneous *very* preterm delivery (< 34 weeks) and risk ratios comparing COVID-19 positive vs. negative and severe vs. mild/moderate.

|  | Negative | Positive | Positive vs. negative | Mild/moderate | Severe | Severe vs. mild/moderate |
| --- | --- | --- | --- | --- | --- | --- |
| Week 20 | 1.5% (1.2, 1.7) | 1.7% (1.1, 2.3) | 1.1 (0.7, 1.6) | 1.7% (1.1, 2.3) | 2.0% (1.0, 3.3) | 1.2 (0.7, 1.8) |
| Week 21 | 1.5% (1.2, 1.7) | 1.6% (1.1, 2.2) | 1.1 (0.7, 1.6) | 1.6% (1.1, 2.2) | 2.1% (1.0, 3.3) | 1.3 (0.8, 1.9) |
| Week 22 | 1.4% (1.2, 1.7) | 1.6% (1.1, 2.2) | 1.1 (0.7, 1.6) | 1.6% (1.0, 2.2) | 2.2% (1.1, 3.5) | 1.4 (0.8, 2.0) |
| Week 23 | 1.4% (1.2, 1.7) | 1.6% (1.1, 2.3) | 1.1 (0.8, 1.6) | 1.6% (1.0, 2.2) | 2.3% (1.1, 3.6) | 1.4 (0.8, 2.2) |
| Week 24 | 1.4% (1.2, 1.7) | 1.6% (1.1, 2.3) | 1.2 (0.8, 1.7) | 1.6% (1.0, 2.2) | 2.5% (1.2, 3.8) | 1.5 (0.9, 2.3) |
| Week 25 | 1.4% (1.1, 1.6) | 1.7% (1.1, 2.2) | 1.2 (0.8, 1.7) | 1.6% (1.1, 2.2) | 2.6% (1.3, 4.0) | 1.6 (1.0, 2.5) |
| Week 26 | 1.3% (1.1, 1.6) | 1.7% (1.1, 2.2) | 1.3 (0.9, 1.7) | 1.6% (1.1, 2.1) | 2.8% (1.4, 4.3) | 1.8 (1.0, 2.7) |
| Week 27 | 1.3% (1.1, 1.5) | 1.7% (1.1, 2.2) | 1.3 (0.9, 1.8) | 1.6% (1.1, 2.1) | 3.0% (1.5, 4.6) | 1.9 (1.1, 2.9) |
| Week 28 | 1.2% (1.0, 1.5) | 1.6% (1.1, 2.2) | 1.3 (0.9, 1.8) | 1.5% (1.1, 2.1) | 3.1% (1.6, 4.8) | 2.0 (1.1, 3.1) |
| Week 29 | 1.2% (1.0, 1.4) | 1.6% (1.1, 2.1) | 1.4 (1.0, 1.8) | 1.5% (1.0, 2.0) | 3.1% (1.6, 4.9) | 2.1 (1.2, 3.3) |
| Week 30 | 1.1% (0.9, 1.3) | 1.4% (1.0, 1.9) | 1.4 (1.0, 1.8) | 1.3% (0.9, 1.8) | 3.0% (1.6, 4.8) | 2.3 (1.2, 3.6) |
| Week 31 | 0.9% (0.8, 1.1) | 1.2% (0.9, 1.6) | 1.3 (0.9, 1.8) | 1.2% (0.8, 1.6) | 2.8% (1.5, 4.5) | 2.4 (1.3, 4.0) |
| Week 32 | 0.8% (0.6, 0.9) | 1.0% (0.7, 1.3) | 1.3 (0.9, 1.7) | 0.9% (0.6, 1.2) | 2.3% (1.2, 3.7) | 2.6 (1.4, 4.3) |
| Week 33 | 0.5% (0.4, 0.5) | 0.6% (0.4, 0.8) | 1.2 (0.9, 1.6) | 0.5% (0.4, 0.7) | 1.4% (0.7, 2.3) | 2.7 (1.4, 4.7) |

Table S10: Estimates of standardized risks of induced *very* preterm delivery (< 34 weeks) and risk ratios comparing COVID-19 positive vs. negative and severe vs. mild/moderate.

|  | Negative | Positive | Positive vs. negative | Mild/moderate | Severe | Severe vs. mild/moderate |
| --- | --- | --- | --- | --- | --- | --- |
| Week 20 | 0.5% (0.4, 0.7) | 0.8% (0.4, 1.2) | 1.5 (0.8, 2.5) | 0.8% (0.4, 1.2) | 1.5% (0.6, 2.9) | 1.9 (1.0, 3.6) |
| Week 21 | 0.5% (0.4, 0.7) | 0.8% (0.4, 1.2) | 1.5 (0.8, 2.5) | 0.7% (0.4, 1.1) | 1.5% (0.6, 2.9) | 2.0 (1.1, 3.8) |
| Week 22 | 0.5% (0.4, 0.7) | 0.8% (0.5, 1.2) | 1.5 (0.9, 2.5) | 0.7% (0.4, 1.1) | 1.6% (0.7, 3.0) | 2.2 (1.2, 4.0) |
| Week 23 | 0.5% (0.4, 0.7) | 0.8% (0.5, 1.2) | 1.6 (0.9, 2.5) | 0.7% (0.4, 1.1) | 1.7% (0.8, 3.3) | 2.3 (1.3, 4.2) |
| Week 24 | 0.5% (0.4, 0.6) | 0.8% (0.5, 1.2) | 1.6 (1.0, 2.6) | 0.8% (0.4, 1.1) | 1.9% (0.8, 3.5) | 2.5 (1.3, 4.5) |
| Week 25 | 0.5% (0.3, 0.6) | 0.8% (0.5, 1.2) | 1.7 (1.1, 2.7) | 0.8% (0.5, 1.1) | 2.0% (0.9, 3.7) | 2.6 (1.4, 4.8) |
| Week 26 | 0.5% (0.3, 0.6) | 0.9% (0.5, 1.2) | 1.8 (1.1, 2.8) | 0.8% (0.5, 1.1) | 2.2% (1.0, 4.0) | 2.8 (1.5, 5.2) |
| Week 27 | 0.5% (0.3, 0.6) | 0.9% (0.5, 1.2) | 1.9 (1.2, 2.8) | 0.8% (0.5, 1.1) | 2.3% (1.1, 4.2) | 3.0 (1.6, 5.7) |
| Week 28 | 0.4% (0.3, 0.6) | 0.9% (0.5, 1.2) | 2.0 (1.3, 2.9) | 0.8% (0.5, 1.1) | 2.4% (1.2, 4.3) | 3.2 (1.7, 6.1) |
| Week 29 | 0.4% (0.3, 0.5) | 0.8% (0.5, 1.2) | 2.0 (1.3, 2.9) | 0.7% (0.4, 1.0) | 2.5% (1.2, 4.4) | 3.4 (1.8, 6.6) |
| Week 30 | 0.4% (0.3, 0.5) | 0.8% (0.5, 1.1) | 2.0 (1.3, 3.0) | 0.7% (0.4, 1.0) | 2.5% (1.2, 4.3) | 3.6 (1.9, 7.1) |
| Week 31 | 0.3% (0.2, 0.4) | 0.7% (0.4, 1.0) | 2.0 (1.3, 3.0) | 0.6% (0.3, 0.8) | 2.3% (1.2, 4.0) | 3.9 (2.0, 7.8) |
| Week 32 | 0.3% (0.2, 0.3) | 0.5% (0.3, 0.8) | 2.0 (1.2, 2.9) | 0.5% (0.2, 0.7) | 1.9% (1.0, 3.3) | 4.1 (2.1, 8.5) |
| Week 33 | 0.2% (0.1, 0.2) | 0.3% (0.2, 0.5) | 1.9 (1.1, 2.8) | 0.3% (0.1, 0.4) | 1.2% (0.6, 2.1) | 4.4 (2.2, 9.2) |

Table S11: Distribution of symptoms and healthcare-related actions across levels of COVID-19 severity.

|  | COVID -19 severity | | | |
| --- | --- | --- | --- | --- |
|  | Mild, N = 2,1041 | Moderate, N = 2,6621 | Severe, N = 2931 | Overall, N = 5,0591 |
| Abdominal pain | 166 (7.9%) | 458 (17%) | 74 (25%) | 698 (14%) |
| Diarrhea | 415 (20%) | 933 (35%) | 104 (35%) | 1,452 (29%) |
| Nausea or vomiting | 418 (20%) | 1,021 (38%) | 124 (42%) | 1,563 (31%) |
| Cough | 885 (42%) | 1,739 (65%) | 220 (75%) | 2,844 (56%) |
| Sore throat | 820 (39%) | 1,377 (52%) | 141 (48%) | 2,338 (46%) |
| Runny nose | 1,039 (49%) | 1,557 (58%) | 147 (50%) | 2,743 (54%) |
| Difficulty breathing/ shortness of breath | 0 (0%) | 1,635 (61%) | 251 (86%) | 1,886 (37%) |
| Bluish lips or face | 12 (0.6%) | 53 (2.0%) | 13 (4.4%) | 78 (1.5%) |
| Pain/pressure in chest | 98 (4.7%) | 586 (22%) | 104 (35%) | 788 (16%) |
| Pneumonia | 0 (0%) | 83 (3.1%) | 132 (45%) | 215 (4.2%) |
| Abnormal chest X-ray or CT scan | 0 (0%) | 107 (4.0%) | 132 (45%) | 239 (4.7%) |
| Acute respiratory distress syndrome | 0 (0%) | 174 (6.5%) | 88 (30%) | 262 (5.2%) |
| Organ failure | 2 (<0.1%) | 9 (0.3%) | 5 (1.7%) | 16 (0.3%) |
| Loss of smell | 1,487 (71%) | 1,989 (75%) | 209 (71%) | 3,685 (73%) |
| Loss of taste | 1,209 (57%) | 1,763 (66%) | 181 (62%) | 3,153 (62%) |
| Muscle aches | 816 (39%) | 1,668 (63%) | 203 (69%) | 2,687 (53%) |
| Tiredness | 1,327 (63%) | 2,262 (85%) | 259 (88%) | 3,848 (76%) |
| Headache | 169 (8.0%) | 232 (8.7%) | 7 (2.4%) | 408 (8.1%) |
| Sudden confusion | 56 (2.7%) | 198 (7.4%) | 54 (18%) | 308 (6.1%) |
| Any fever | 551 (26%) | 1,080 (41%) | 176 (60%) | 1,807 (36%) |
| High fever | 322 (15%) | 719 (27%) | 143 (49%) | 1,184 (23%) |
| Other symptoms | 303 (14%) | 413 (16%) | 31 (11%) | 747 (15%) |
| Called a doctor | 1,057 (50%) | 1,308 (49%) | 134 (46%) | 2,499 (49%) |
| Visited a clinic/hospital | 61 (2.9%) | 1,165 (44%) | 124 (42%) | 1,350 (27%) |
| Visited the ER | 42 (2.0%) | 902 (34%) | 66 (23%) | 1,010 (20%) |
| Was hospitalized | 10 (0.5%) | 67 (2.5%) | 258 (88%) | 335 (6.6%) |
| Needed respiratory assistance/ventilation/ECMO | 0 (0%) | 0 (0%) | 87 (30%) | 87 (1.7%) |
| Admitted into intensive care (ICU) | 0 (0%) | 0 (0%) | 66 (23%) | 66 (1.3%) |
